# Book Notices

**Published:** 1899-08

**Authors:** 


### BOOK NOTICES.

THE SEXUAL INSTINCT: Its Use and Dangers as Affecting Heredity and Morals. By James Foster Scott, B. A., M. D., C. M. New York: E. B. Treat & Co., 241-243 West Twenty-third Street. 1899. Price, $2.00.

This exceedingly valuable book, valuable for the lessons it teaches, for the advice it gives, for the good it is intended to do, should be in the hands of all men and women whether in or out of the medical profession.

It is an earnest and eloquent protest against the sexual abuses that shame the individual man and woman, disgrace the community, and lead to the terrible crimes which threaten the very foundations of society.

It treats of the sexual instinct, its uses and its influence. It describes clearly the physiology of the sexual life; it gives "a proper calculation of the consequences of impurity from the personal standpoint;" it pays a high tribute to woman, whom it calls the "sweetest of God's creatures;"

it treats of the influences which incite to sexual morality; it devotes a chapter to prostitution and its causes; it inveighs bitterly against criminal abortion; it unfolds the disasters of gonorrhœa and syphilis; and it warns against Onanism and other perversions.

As the author well says in his preface, "This book contains much plain talking, for which I offer no defence." Its design "to furnish the non-professional man with a sufficiently thorough knowledge of matters pertaining to the sexual sphere—knowledge which he cannot afford to be without."

In defence of his motive for writing the book, which is to, in some measure, reduce the amount of sexual depravity, the author says:

"It is commonly said that it is a hopeless task to turn the stream of the sexual activities into orderly channels. So also is it a hopeless task to do away with murder, theft, drunkenness, lying, and other prevalent misdeeds. Evils, however, can be mitigated, if not cured, if we subject them to philosophical analysis, which may suggest remedies."

"Painful as it is to treat subjects so repulsive, a man cannot choose his duty, nor can he honestly evade it. Therefore, knowing no other book of like character, I present this as the best effort of which I am at present capable for the preservation of the individual and the welfare of the race."

With this short notice and the quotations of the author's own statements of his reasons for writing the book, we commend it to the consideration of the appreciative readers of this journal.

The Practical Dog Book for both the professional and amateur fancier. We have received from the Associated Fanciers, 400 N. Third street, Philadelphia, Pa., a copy of their Dog Buyers' Guide. It contains a finely-executed colored frontispiece; well drawn engravings of nearly every breed of dog, and all kinds of dog furnishing goods. We should judge that the book has cost a great deal more to produce than the price asked—15 cents—and we would advise all of our readers who are interested in dogs to send for the book.

A Book on Poultry, containing 116 pages, a beautiful lithographic plate of a group of different fowls in natural colors, engravings of all kinds of land and water poultry, descriptions of the breeds, plans for poultry houses, how to manage an incubator, all about caponizing, and the value of different breeds. It will be mailed to any of our readers for 15 cents by the Associated Fanciers, 400 North Third street, Philadelphia, Pa.

THE TOY DOG. A copy of Mr. John E. Diehl's latest book on the Toy Dog has just been submitted to us for criticism. We can speak of the little volume only in terms of the highest praise. The author, who was recognized for years as an authority on domestic pets of all kinds, has evidently put his best efforts on his last production, so that this becomes almost invaluable to all who admire, or intend to provide themselves with a toy dog. The book has been published by the Associated Fanciers, 400 North Third street, Philadelphia, Pa., who offer to mail it to any address on receipt of 25 cents, preferably in postage stamps.

